# Error in Key Points

**DOI:** 10.1001/jamanetworkopen.2019.16430

**Published:** 2019-11-06

The Original Investigation titled “Assessment of Accuracy of an Artificial Intelligence Algorithm to Detect Melanoma in Images of Skin Lesions,”^1^ published October 16, 2019, included an error in the Key Points section. The area under the receiver operating characteristic curve that appeared corresponded to the results for digital single-lens reflex camera images, whereas the specificity corresponded to the results for iPhone images. The area under the receiver operating characteristic curve for those images was 95.8%. This article has been corrected.^1^
